# The Duties of Dermatologists During COVID-19 Pandemic in Turkey: Results of a Nationwide Survey

**DOI:** 10.14744/SEMB.2021.67750

**Published:** 2021-12-29

**Authors:** Muazzez Cigdem Oba, Kursat Goker

**Affiliations:** Department of Dermatology and Venereology, Health Sciences University, Sancaktepe Sehit Prof. Dr. Ilhan Varank Training and Research Hospital, Istanbul, Turkey

**Keywords:** Coronavirus, dermatology, pandemic

## Abstract

**Objectives:**

The coronavirus disease 2019 (COVID-19) has led to a reorganization of health services throughout many countries. In this study, we aimed to get an overview of the duties of the dermatologists during COVID-19 pandemic in Turkey. In light of the results, we aimed to determine the aspects of Turkish dermatology practice which might require innovation.

**Methods:**

Dermatologists across Turkey were asked to fill in an online 11-item questionnaire survey, investigating their duty/duties (dermatology outpatient and inpatient clinics, pandemic outpatient and inpatient clinics, emergency, etc.) month by month during March–June 2020.

**Results:**

A total of 217 dermatologists participated in the survey. Vast majority (91–98%) of the participants reported that they performed dermatology outpatient visits. While 41.5–56.2% of participants were redeployed to pandemic inpatient clinics, 12.9–29% were mobilized to pandemic outpatient clinics. Each month, at least 90% of the residents that participated in the questionnaire reported that they were recruited to pandemic inpatient clinics.

**Conclusion:**

As the impact of COVID-19 pandemic is ongoing in Turkey, these data should be taken into consideration to rapidly implement new measures in Turkish dermatology practices such as a referral system for dermatology outpatient visits to equitably distribute dermatology services, widespread use of telemedicine, and virtual educations of residents.

The coronavirus disease 2019 (COVID-19) caused by the severe acute respiratory syndrome coronavirus 2 has led to a reorganization of health services throughout many countries. Junior doctors as well as physicians of various specialties were recruited to intensive care units, COVID-19 wards, emergency units, etc.^[1-5]^ In Turkey, many hospitals mobilized physicians from all of specialties to take part in the care of patients with COVID-19, including the authors’.^[6]^ In addition, non-emergent outpatient visits were suspended in many countries.^[7-9]^

In this study, we aimed to get an overview of the role of the dermatologists during COVID-19 pandemic in Turkey. In light of the results, we aimed to determine the aspects of Turkish dermatology practice which might require innovation.

## Methods

Dermatologists across Turkey were asked to fill in an online 11-item questionnaire survey, composed on Google Forms. The survey was distributed to the dermatologists across Turkey through email groups, social media, and WhatsApp groups. The participants were allowed to respond the questionnaire from beginning of July 2020 till August 15.

The questions included the age and sex of the participants, their affiliation and academic degree, the city where they worked, and their duty/duties (dermatology outpatient and inpatient clinics, pandemic outpatient and inpatient clinics, emergency, etc.) month by month during March–June 2020. In the last two questions, daily number of dermatology outpatients examined and number of monthly shifts in pandemic inpatient clinics were asked. All questions were obligatory.

The study was approved by both the local review board (approval number: 116.2017.183 approval date: July 02, 2020) and Turkish Ministry of Health.

### Statistical Analysis

Descriptive statistics were reported as mean, minimum, and maximum for continuous data; as count and percentage for categorical data. Chi-square test and Bonferroni correction were used to analyze categorical data and make multiple comparisons between groups. Statistical analyses were performed using the Statistical Package for the Social Sciences (SPSS, Chicago, IL, USA) version 21.0. P<0.05 was considered statistically significant.

## Results

### Descriptive Statistics

A total of 217 responses were obtained from dermatologists across Turkey. Dermatologists of all ages, from residents to senior dermatologists, participated in the survey (age range: 27–70). Majority of them (68.7%) were women. Vast majority of the participants were working in governmental hospitals including research and training hospitals (28.1%) and public hospitals (25.3%) followed by university hospitals (24.4%) and private hospitals (13.8%). Although respondents were from nearly all cities of Turkey, dermatologists from three biggest cities of Turkey dominated (34.6%, 10.1%, and 6% for Istanbul, Ankara, and Izmir, respectively). Academic degrees of participants were specialist (66.4%), resident (18.9%), associate professor (8.3%), and professor (6.5%). Demographic data of participants are shown in Table 1.

**Table 1.** Demographic data of participants

**Parameters**	**Number (%)**
Sex	
Male	68 (31.3)
Female	149 (68.7)
Age	
27–30	54 (24.9)
31–40	88 (40.6)
41–50	41 (18.9)
51–60	27 (12.4)
>60	7 (3.2)
Affiliation	
Public hospital	55 (25.3)
Public research and training hospital	61 (28.1)
University hospital	53 (24.4)
Private hospital	30 (13.8)
Private practice	12 (5.5)
Public city hospital	6 (2.8)
Academic degree	
Resident	41 (18.9)
Specialist	144 (66.4)
Associate professor	18 (8.3)
Professor	14 (6.5)
Region	
Marmara	96 (44.2)
Aegean	22 (10.1)
Black sea	20 (9.2)
Central Anatolia	43 (19.8)
East Anatolia	7 (3.2)
Southeast Anatolia	17 (7.8)
Mediterranean	12 (5.5)

### Dermatology Outpatient Visits

**Figure 1.**
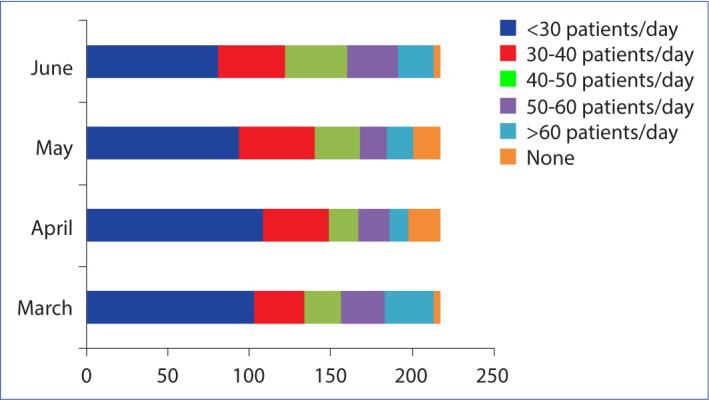
Monthly distribution of participants working in dermatology outpatient clinics and number of daily visits performed.

Vast majority of the participants reported that they performed dermatology outpatient visits. In March and June, 98% of participants worked in outpatient clinics. The percentage was reduced to 91% and 93% in April and May, respectively. Figure 1 shows distribution of participants working in dermatology outpatient clinics and number of daily visits performed, on monthly basis.

### Dermatology Inpatient Clinics

In March, 21.2% of participants stated that they worked in dermatology inpatient clinics. This percentage dropped to 12.4% in April, though not significantly (p=0.06). In May and June, 17% and 24.4% of participants stated that they worked in dermatology wards. A statistically significant difference was detected between April and June (p=< .0.001).

### Pandemic Inpatient Clinics

**Figure 2.**
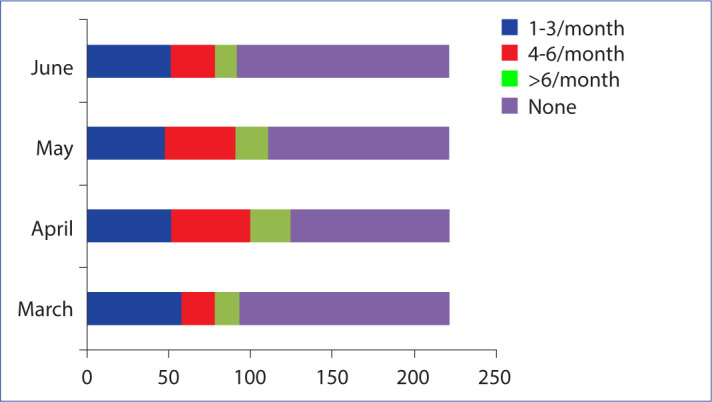
Monthly distribution of participants involved in pandemic inpatient care and number of monthly shifts.

In March, 42.4% of the participants were recruited to pandemic inpatient clinics. This percentage significantly increased in April as 56.2% of dermatologists stated that they worked in pandemic inpatient clinics (p=0.001). In May and June, 50.2% and 41.5% of participants reported working in pandemic inpatient clinics. Statistical difference was also found in the percentage of participants caring for COVID-19 inpatients between April and June (p=< 0.001). Most of the participants who were recruited in pandemic inpatient clinics indicated having one to three shifts per month. The monthly distribution of participants involved in pandemic inpatient care along with number of monthly shifts is shown in Figure 2. Participants recruited in pandemic inpatient clinics during March–June 2020 were all among public staff of research and training hospitals, public hospitals, and university hospitals. Participants working in private hospitals or private practice indicated that they did not take part in pandemic inpatient care except for two participants working in private hospital who stated working in pandemic inpatient care in April. Analysis according to academic degree revealed residents and specialist to form the vast majority of participants recruited to pandemic inpatient clinics. During four months, only 2.8–6.5% of participants recruited to COVID wards were associate professor and professors. However, each month, at least 90% of the residents that participated in the questionnaire reported that they were recruited to pandemic inpatient clinics. During April 2020, 26.9% of participants reported that they worked solely in pandemic inpatient clinics. This ratio decreased to 17.6% and 3.1% of participants in May and June, respectively.

### Pandemic Outpatient Clinics

Pandemic outpatient clinics were created throughout Turkey, to function as COVID-19 screening outpatient clinics. Patients with fever, cough, and dyspnea were firstly examined in these clinics so as to isolate these potential cases from other patients seeking medical care. In March, 12.9% of respondents reported that they worked in pandemic outpatient clinics. A surge in the number of participants (29%) that worked in COVID-19 screening outpatient clinics was seen in April, with a significant statistical difference between March and April (p=< 0.001). In May and June, 24.4% and 15.2% of participants reported that they worked in pandemic outpatient clinics. Statistically, the difference between March and May (p=< 0.001) and April and June (p=< 0.001) was also significant.

## Discussion

This study demonstrated that majority of the dermatologists working in Turkey were actively involved in the care of COVID-19 patients as well as pursuing dermatology clinical practices. Considering there were 2351 actively working dermatologists in Turkey during the study period, results of our survey with 217 participants have a 95% confidence interval and a margin error of 6.3%.

Across the globe, the COVID-19 pandemic has had its impact on the dermatology practice. Number of patients seen in outpatient clinics and number of surgeries performed significantly decreased.^[8,10,11]^ In various countries, dermatologists were involved in the fight against COVID-19 pandemic. In the US, 31.9% of dermatology residents were redeployed to non-dermatology services including emergency department, inpatient wards, and intensive care units.^[12]^ Dermatology staff of Penn Medicine, US, was redeployed to assess and manage COVID-19 results of their health care system’s emergency department and ambulatory test sites.^[13]^ In Italy, Spain, and UK, dermatologists were relocated to care for COVID-19 patients.^[11,14,15]^ In India, dermatologists were not involved in the primary care of COVID cases, however, outpatient clinics were closed in many hospitals and dermatology wards were converted to COVID wards.^[16]^ Hospitalizations in dermatology inpatient clinics were completely or partially suspended in many countries.^[7,16,17]^ Similarly, our survey revealed that the number of participants that worked in dermatology inpatient clinics was significantly reduced in April, as compared to June (p=< 0.001).

In Turkey, the first confirmed COVID-19 case was announced on March 11, 2020. According to the data provided by the Turkish Ministry of Health, the number of confirmed COVID-19 cases was 198,284 from March 11, 2020, till June 28, 2020.^[18]^ Shortly after the first case, Turkish government applied a partial lockdown, closed schools and restaurants, and restricted domestic and abroad travels. However, at June 1, several restrictions were reduced, considered as “normalization steps.”

Kutlu et al. reported a significant decrease in the number of dermatology outpatient visits immediately after the beginning of COVID-19 pandemic in Turkey by comparing the average number of patients examined 10 days before and 10 days after the first COVID-19 case in Turkey. The study showed that, despite the sharp decrease, the number of daily visits did not drop under 39 and the most common five diagnoses were all non-emergent diagnoses such as acne, warts, and seborrheic dermatitis.^[19]^ Another study by Cengiz et al. revealed 390 presentations to one dermatology outpatient clinic in Istanbul during March 11–18, 2020.^[20]^ Tanacan et al. reported a significant decrease (from 1165 to 717) in number of hospital admissions of a tertiary dermatology clinic by comparing the number of admissions during March–May 2019 to that of 2020.^[21]^ A multicenter study conducted between January 12, 2020, and May 12, 2020, found 77% decrease in daily hospital applications.^[22]^ However, our data showed that a substantial number of dermatology outpatient clinic visits were performed during March–June 2020 despite the pandemic. This finding does not contradict the literature and it is attributable to the fact that dermatology outpatient clinics were overcrowded in the pre-pandemic period in Turkey. In recent years, Turkish health policies have been aiming to increase patient satisfaction.^[23]^ During COVID-19 pandemic, measures should have been taken to suspend all non-emergent visits^[24]^ to limit both patient-patient and patient-physician COVID transmission. However, this was not the case in Turkey even during the early pandemic, as our findings have shown that each month, half of dermatologists reported examining more than 30 patients per day. In addition, Turkish health-care system does not require patients to have a referral to make an appointment to dermatologists and vast majority of the busy dermatology outpatient visits performed are non-emergent. During COVID pandemic, the Turkish health ministry has only recommended, but not obliged, the patients to visit their general practitioner before applying to dermatology outpatient clinics. We think this approach, along with the decreased dermatology staff due to redeployment of dermatologists to COVID wards, might have led to significant delays in emergent dermatology visits. In addition, restrictions for elderly (>65 years) people such as the ban of using public transport might also have contributed to uneven distribution of emergent dermatology services.

Considering that dermatologists are physicians first, they may be recruited to COVID wards, triage stations, and even ICUs, if need occurs.^[15,25,26]^ Our study showed that 41.5–56.2% of participants were redeployed to pandemic inpatient clinics. Across 4 months, the percentage of participants that were mobilized to COVID inpatient clinics and outpatient clinics changed. These changes were in accordance with the rate of COVID cases in Turkey as the first case was in March 11, the rapid rise in April and May, and a slowing down in June. Of note, a strikingly high percentage of at least 90% of residents that participated in the questionnaire stated that they were recruited to pandemic inpatient clinics during March–June 2020. This might have significantly impaired specialty education of dermatology residents in Turkey. E-learning programs were created for medical students across all universities in Turkey.^[27]^ To overcome the disruption in resident education, implementation of virtual lectures and supervised telemedicine visits that incorporate residents should also be planned, as accomplished in various countries.^[8,28-30]^

Limitations of our study include the recall bias, undercoverage of associate professors and professors, and the lack of a question asking whether the redeployment was volunteered or of necessity. However, all of dermatologists that were redeployed to COVID-19 inpatient and outpatient clinics were staff of public hospitals, except two. This finding supports the assumption that the mobilization of dermatologists was mostly because of health policies.

## Conclusion

Our findings indicate that the COVID-19 pandemic has significantly affected the daily clinical practice of dermatologists in Turkey. Considering the ongoing impact of COVID-19 pandemic in many countries including Turkey, these data should be taken into consideration to rapidly implement new measures such as a referral system for dermatology outpatient visits to equitably distribute dermatology services, widespread use of telemedicine, and virtual educations of residents.

### Disclosures

**Ethics Committee Approval:** This study was approved by the ethşcs committee of Health Sciences University, Süreyyapasa Chest Diseases and Thoracic Surgery Research and Training Hospital (no: 116.2017.183, approval date: July 2, 2020).

**Peer-review:** Externally peer-reviewed.

**Conflict of Interest:** None declared.

**Authorship Contributions:** Concept – K.G.; Design – M.C.O., K.G.; Supervision – K.G.; Materials – M.C.O., K.G.; Data collection &/or processing – K.G.; Analysis and/or interpretation – M.C.O.; Literature search – M.C.O.; Writing – M.C.O., K.G.; Critical review – M.C.O.
